# Dual Stabilization of *S*‑Adenosylmethionine for Enzymatic DNA Labeling

**DOI:** 10.1021/jacsau.6c00154

**Published:** 2026-03-12

**Authors:** Jonas Bucevičius, Ru̅ta Gerasimaitė, Gražvydas Lukinavičius

**Affiliations:** Chromatin Labeling and Imaging Group, Department of NanoBiophotonics, 28282 Max Planck Institute for Multidisciplinary Sciences, Am Fassberg 11, 37077 Göttingen, Germany

**Keywords:** *S*-Adenosylmethionine, cofactor engineering, proline derivatives, methyltransferases, DNA labeling

## Abstract

*S*-Adenosyl-l-methionine (AdoMet) analogues are a powerful tool for site-specific biomolecular labeling via methyltransferase (MTase)-catalyzed transfer reactions. However, their utility is often limited by their poor chemical stability under enzymatic reaction conditions. Here, we report a new class of stabilized AdoMet analogues, featuring a conformationally constrained proline side chain in place of homoalanine. This substitution inhibits intramolecular cyclization, which is a major decomposition pathway. Combination with selenonium modification, which suppresses depurination, yields analogues with up to a 90-fold increase in half-life relative to AdoMet. These cofactors retain activity with DNA MTases and allow sequence-specific labeling of plasmid DNA using both two- and single-step approaches with fluorescent dyes.

## Graphical abstract

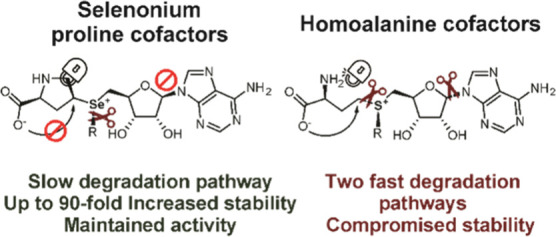

## Introduction

*S*-Adenosyl-l-methionine (AdoMet) is one of the most ubiquitous cofactors present in all living organisms, playing a central role in a wide array of biochemical reactions and intracellular regulatory pathways.^1^ Methyltransferases (MTases) catalyze S_N_2-like reactions between nucleophiles in biomolecules and the electrophilic carbon next to the sulfonium center of AdoMet.^2^ Transfer of larger substituents from AdoMet analogues is usually hampered; however, the reaction efficiency can be restored by using synthetic analogues bearing sp- or sp^2^-hybridized carbon at the β-position to the sulfonium center,^3–6^ which stabilizes the intermediate state and increases the transfer rate through a secondary orbital overlap effect. The transfer of larger groups from synthetic AdoMet-analogues by native or engineered MTases is exploited in late-stage modification of complex organic molecules,^7,8^ biomolecule enrichment,^9,10^ labeling,^11^ sequencing,^9,12,13^ and optical genome mapping.^14^ However, the AdoMet and especially its activated analogues are chemically unstable under enzymatic reaction conditions. They decay mainly via two main pathways: intramolecular cyclization, leading to the formation of homoserine lactone and 5′-deoxy-5′-(alkylthio)­adenosine or its derivatives, and depurination, resulting in adenine base and homocysteine sulfonium ribose derivatives^15^ (Figure 1).

**Figure 1:**
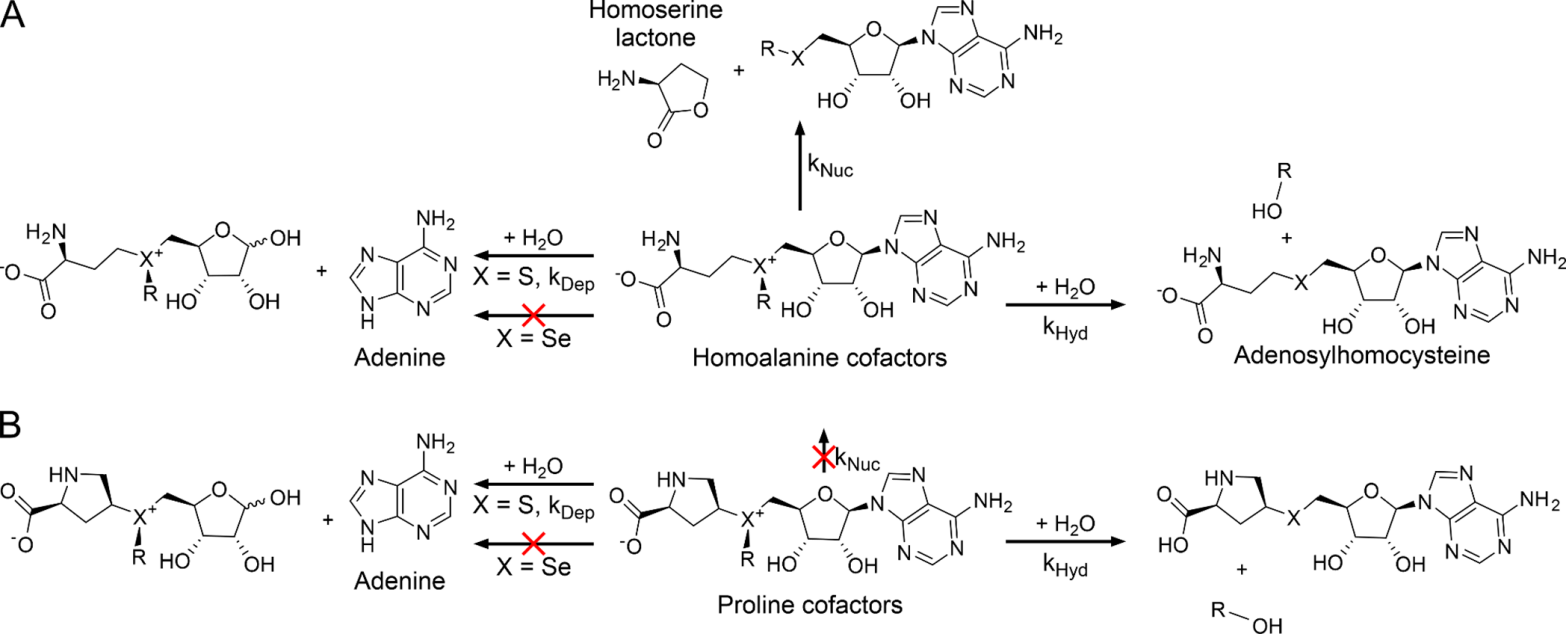
Major decomposition pathways of homoalanine (A) and proline (B) cofactors. Decomposition at physiological pH of AdoMet and its analogues can proceed via multiple pathways depending on the structure of the transferable side chain (R) and chalcogenonium center (X). Dominating decay products allow the estimation of each pathway rate: *k*_Nuc_, intramolecular nucleophilic cyclization; *k*_Dep_, depurination; and *k*_Hyd_, hydrolysis. Replacement of homoalanine with proline introduces steric hindrance and effectively eliminates intramolecular cyclization.

Since the first examples of methyltransferase-directed transfer of activated groups (mTAG),^3–5^ there have been only a few reports of chemically more stable designs of AdoMet analogues. However, the enzymatic activity was either lost^16^ or maintained only with promiscuous small-molecule transferases, such as DnrK, COMT, PNMT, and NNMT, and none of them maintained activity with DNA MTases.^8,17,18^ Herein, we report an AdoMet analogue design, wherein the homoalanine side chain is replaced with a conformationally rigid proline side chain, eliminating the intramolecular cyclization decay pathway. When combined with sulfur-to-selenium substitution, which reduces depurination at higher pH, the modified cofactors display up to 90-fold enhanced stability under enzymatic reaction conditions. We demonstrate that these cofactor analogues maintain enzymatic activities with the widely used thermophilic adenine DNA MTase TaqI and an engineered cytosine DNA MTase HhaI.

## Results and Discussion

### Design, Synthesis, and Initial Enzymatic Activity Testing

We hypothesized that replacing the homoalanine side chain with a proline residue would inhibit the intramolecular cyclization pathway by restricting access to a favorable reactive conformation. Proline contributes an intrinsic stereocenter at the α-carbon (Cα), giving rise to l and d configurations, while the chalcogenonium center constitutes a second stereogenic element, affording *R* and *S* diastereomers. In addition, the relative *cis*/*trans* arrangement between the proline carboxyl group and the thioadenosine backbone further defines the overall three-dimensional architecture. All combinations of these stereochemical elements were prepared (Scheme 1 and Figures S1–S4) and evaluated for activity with the DNA methyltransferases TaqI and the engineered HhaI Q82A/Y254S/N304A variant, engineered to work efficiently with activated AdoMet analogues.^19^ The synthesis commenced from enantiomerically pure *N*-Boc-4-hydroxyproline methyl esters (Scheme 1). The hydroxy group was converted to a mesylate, followed by S_N_2-type nucleophilic substitution with either potassium thioacetate (KSAc) or potassium selenocyanate (KSeCN), affording intermediates **5**–**9**. Subsequent generation of the corresponding thiolate or selenolate anions enabled nucleophilic substitution of either 5′-tosyladenosine or 5′-chloro-7-deazaadenosine to yield compounds **10**–**15**. After ester hydrolysis and Boc deprotection, the resulting compounds **16**–**21** were alkylated by using mesylates (**SI-1**–**SI-3**) or triflates (**SI-4** and **SI-5**) to furnish diastereomeric mixtures of final cofactors **22**–**36**. The obtained diastereomeric mixtures were separated by preparative reversed-phase HPLC (Figure S5). In the case of cofactors with methyl transferable groups (**28ab**–**30ab**), we were unable to separate diastereomers; therefore, these compounds were tested and used as two diastereomer mixtures. In all other cases, separations were successful.

**Scheme 1:**
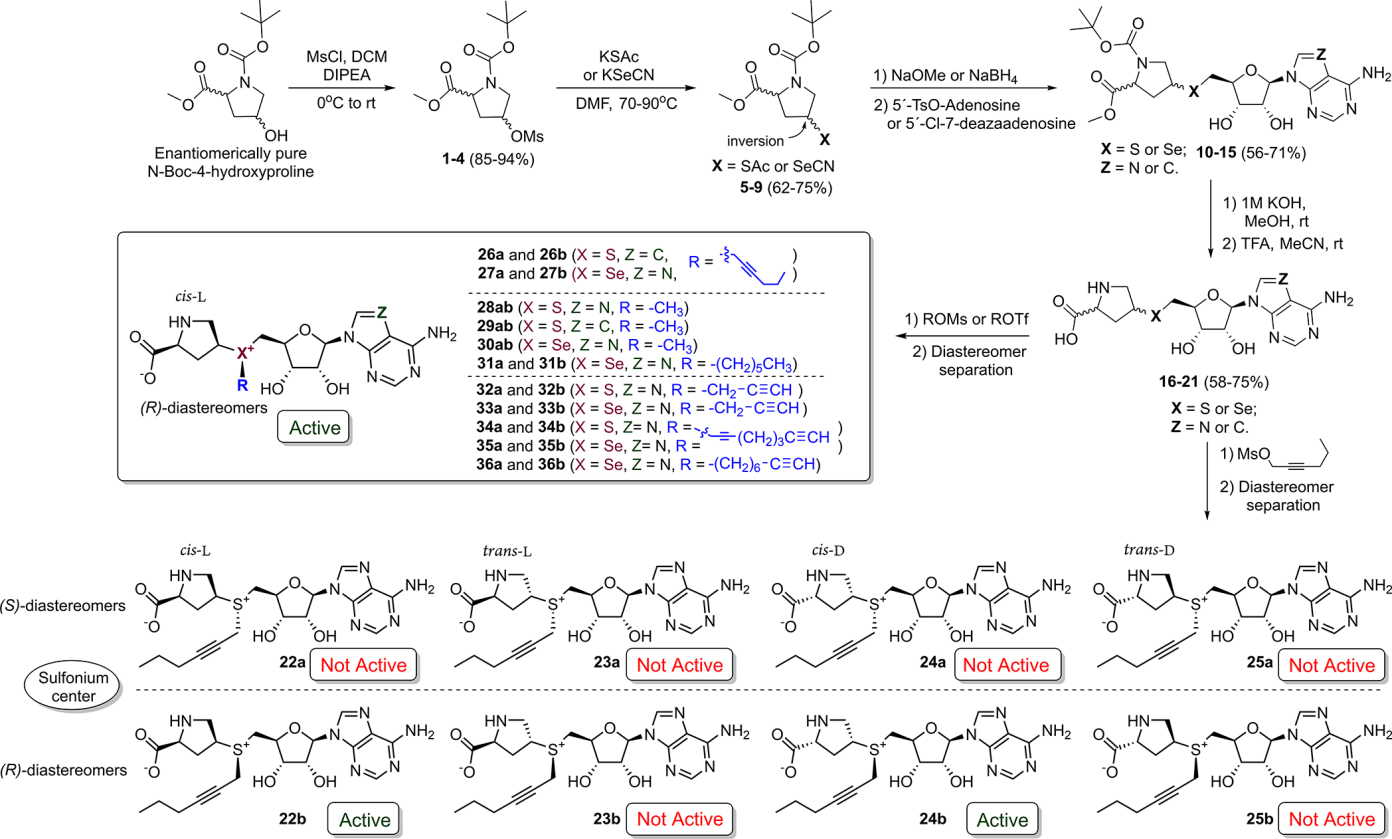
Synthesis Scheme of Proline Cofactors **22**–**36** ^a^

a All possible stereoisomers of the proline-derived cofactors are shown, including the α-carbon (Cα) isomers (l/d configuration), both *S* and *R* diastereomers and *cis*/*trans* arrangement of the proline ring with respect to the cofactor backbone. The synthesis was initiated from enantiomerically pure *N*-Boc-4-hydroxyproline. Only the *R* diastereomers with *cis*-l and *cis*-d configurations are enzymatically active, whereas *trans* isomers and *S* diastereomers are inactive.

The activity of the new AdoMet analogues in DNA MTase-catalyzed reactions was evaluated using a DNA protection assay, which relies on the inability of restriction enzymes to cleave DNA-containing modified bases within their recognition sequences (Figure S6). Cofactors **22a**–**25a** and **22b**–**25b**, bearing a transferable hex-2-ynyl side chain (Scheme 1), were tested with the thermophilic DNA adenine-N6 methyltransferase TaqI.^3^ Among these, only the *cis*-l-proline and *cis*-d-proline configuration analogues (**22b** and **24b)** exhibited enzymatic activity (Scheme 1 and Figure S7). By analogy with the active stereoisomer of AdoMet, the sulfonium or selenonium center in these cofactors is presumed to adopt the *R* configuration. We further assume that all other cofactors exhibiting longer retention times (**26b**, **27b**, **31**–**36b**) during preparative HPLC purification have the same *R* configuration. The *cis*-l-proline analogue **22b** was also active with the M.HhaI Q82A/Y254S/N304A variant (Figure S8). On this basis, all subsequent analogues were designed around the *cis*-l-proline scaffold as a more promising variant.

For comparative analysis, sulfonium- and selenonium-based cofactors (**37b**–**40b**), bearing the canonical homoalanine side chain and a series of transferable groups (methyl, propargyl, and hex-2-ynyl), were prepared (Figure 2A). The synthesis involved alkylation of *S*-adenosylhomocysteine or *Se*-adenosylhomocysteine with the corresponding alkyl mesylates or methyl triflate (Figure S9), followed by preparative HPLC purification.

**Figure 2:**
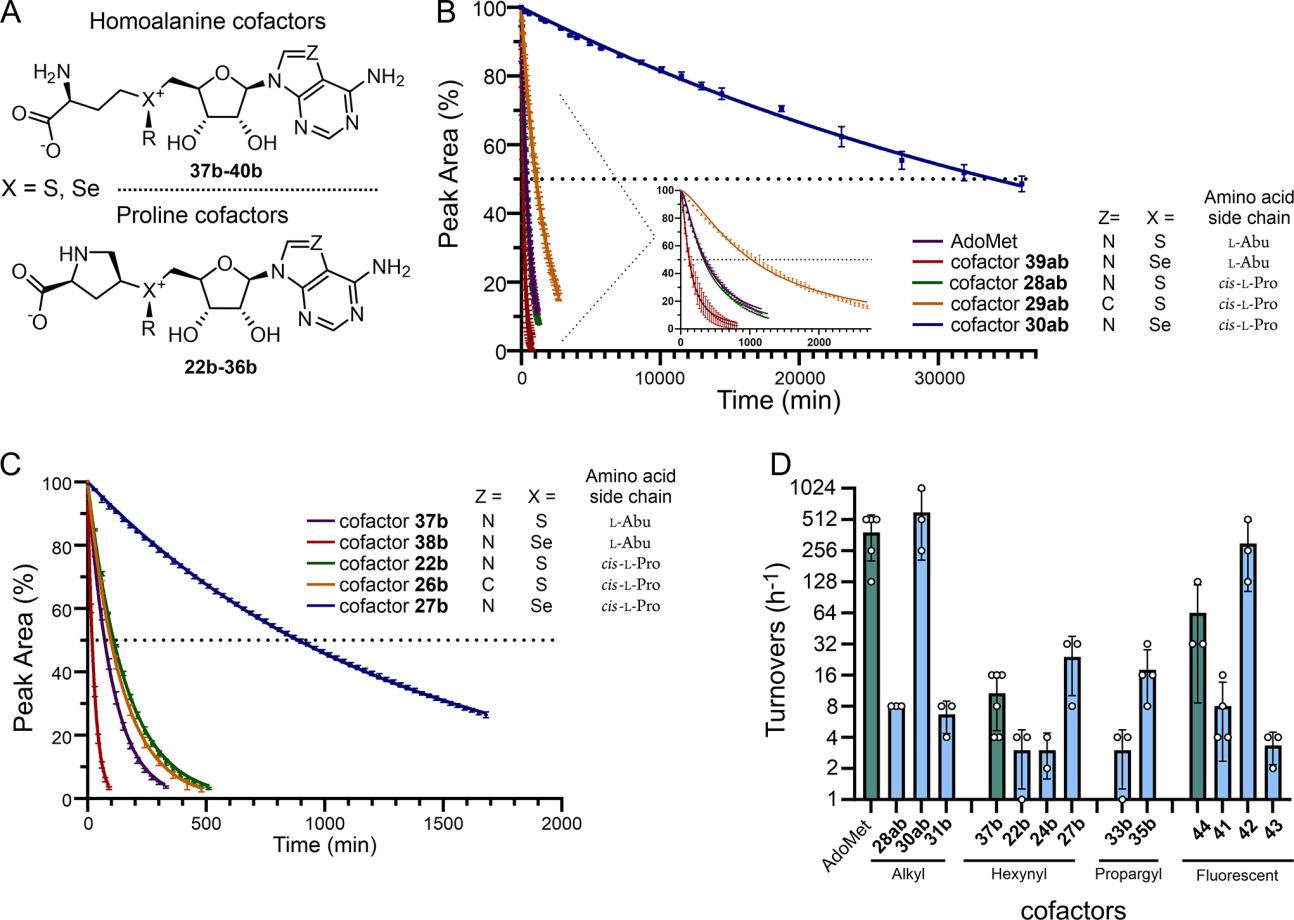
Stability and activity of homoalanine and proline cofactors. (A) General structures of the homoalanine and proline cofactors. Degradation kinetics of AdoMet or its analogues containing transferable methyl (B) or hex-2-ynyl groups (C) in 50 mM Tris-HCl (pH 8.0) at 37 °C and *N* = 3. l-Abu, l-homoalanine; *cis*-l-Pro, *cis*-l-proline. (D) Apparent turnover numbers of M.TaqI with homoalanine (green bars) and proline (blue bars) cofactors measured via DNA protection assay in 50 mM Tris-MOPS (pH 8.0) at 65 °C and *N* ≥ 3.

For single-step labeling of DNA with fluorescent dyes, we synthesized *cis*-l-proline cofactors **41**–**43** by conjugating terminal alkyne-bearing intermediates **34b**–**36b** with 4-MeSiR-PEG_4_-N_3_ via the CuAAC reaction, introducing a fluorescent reporter directly into the transferable moiety in 30–67% yields. For comparison, a classical homoalanine-based cofactor analogue **44** was prepared similarly from AdoHcyN_3_ ^5^ and 4-MeSiR-PEG_4_-alkyne (Figure S10).

### Stability, Degradation, and Kinetics

The chemical stability and decomposition products of the prepared cofactors were evaluated in a 50 mM Tris–HCl buffer (pH 8.0) at 37 °C (Figure 2). Time-dependent decomposition analysis revealed behavior consistent with first-order decay kinetics, indicative of (pseudo)­unimolecular decomposition processes (Figures 2 and S11–S15 and Table 1). AdoMet exhibited a half-life of 366 min (Figure 2B), with equal contributions from intramolecular cyclization and depurination pathways (Table 1 and Figure S16). Substitution of the sulfonium center with selenonium (SeAdoMet^15^ **39ab**) suppressed the depurination pathway (Figure S16) but accelerated intramolecular cyclization due to the higher nucleofugality of diselenyl ethers,^20^ resulting in a reduced half-life of 133 min. We noticed that the decomposition product, methylselenoadenosine, was partially oxidized to methylselenoxideadenosine under analytical conditions,^21^ and this oxidation was taken into account in the half-life and degradation reaction rate calculations. Importantly, the selenium incorporation completely suppressed depurination, as the formation of a selenium ylide intermediate requires higher than physiological pH values.^15,22^

**Table 1:** Half-Lives and Decay Rate Constants of the Studied Cofactors^a^

cofactor	amino acid chain	X	Z	–R	*t*_1/2_ (min) [95% CI]^b^	*k*_total_ (×10^–^^5^ s^–1^)	*k*_Nuc_ (×10^–^^5^ s^–1^)	*k*_Dep_ (×10^–^^5^ s^–1^)	*k*_Hyd_ (×10^–^^5^ s^–1^)
AdoMet	l-Abu	S	N	–CH_3_	366 [359, 372]	3.09 ± 0.07	1.30 ± 0.05	1.34 ± 0.06	
SeAdoMet (**39ab**)	l-Abu	Se	N	–CH_3_	133 [127, 139]	8 ± 2	8 ± 2		
cofactor **28ab**	*cis*-l-Pro	S	N	–CH_3_	346 [345, 348]	3.42 ± 0.03		3.35 ± 0.05	
cofactor **30ab**	*cis*-l-Pro	Se	N	–CH_3_	34,012 [33378, 34664]	0.034 ± 0.003			0.027 ± 0.002
cofactor **31b**	*cis*-l-Pro	Se	N	–(CH_2_)_5_CH_3_	73,968 [71017, 77151]	0.015 ± 0.003			0.012 ± 0.004
cofactor **37b**	l-Abu	S	N	–CH_2_C≡C(CH_2_)_2_CH_3_	72 [70, 73]	17.1 ± 0.5	5.5 ± 0.2	2.72 ± 0.09	
cofactor **38b**	l-Abu	Se	N	–CH_2_C≡C(CH_2_)_2_CH_3_	19 [18, 19]	64 ± 3	52 ± 2		
cofactor **22b**	*cis*-l-Pro	S	N	–CH_2_C≡C(CH_2_)_2_CH_3_	110 [108, 111]	11.4 ± 0.4		7.5 ± 0.3	0.059 ± 0.001
cofactor **27b**	*cis*-l-Pro	Se	N	–CH_2_C≡C(CH_2_)_2_CH_3_	888 [885, 891]^b^	1.30 ± 0.02			0.79 ± 0.01
cofactor **33b**	*cis*-l-Pro	Se	N	–CH_2_C≡CH	45 [44, 46]	26.8 ± 0.7			
cofactor **35b**	*cis*-l-Pro	Se	N	–CH_2_C≡C(CH_2_)_3_C≡CH	925 [907, 944]	1.27 ± 0.05			0.58 ± 0.02

a l-Abu, l-homoalanine; *cis*-l-Pro, *cis*-l-proline.

b 95% confidence interval is reported in square brackets.

Replacing the homoalanine side chain with a proline moiety completely inhibited the intramolecular cyclization pathway (Figure S17); however, the depurination rate slightly increased and the analogue **28ab** showed a similar half-life (346 min, 5.8 h) to AdoMet. It is known that the use of a purine isostere, such as 7-deazapurine, can suppress depurination^17^ at physiological pH values due to limited cascade charge redistribution from the formed ylide to carbon at the 7 position. Indeed, compound **29ab** exhibited a markedly extended half-life of ∼17 h, with slow depurination as the dominant degradation pathway (Table S1 and Figure 2B). Remarkably, the selenium-containing cofactor **30ab** displayed complete suppression of both depurination and cyclization (Table 1 and Figures 2B and S17), leading to a half-life of ∼24 days—90-fold greater than that of native AdoMet. Here, the only remaining decomposition route was the slow hydrolysis of the transferable methyl group.

Next, we measured the stabilities of cofactors bearing activated hex-2-ynyl groups. Half-life of homoalanine containing cofactor **37b** was 72 min with a major degradation by intramolecular cyclization and minor by depurination (Figures 2C, S11, and S16). As expected, the corresponding selenonium cofactor **38b** exhibited markedly accelerated degradation, proceeding predominantly via an intramolecular cyclization pathway with a half-life of 19 min (Figures 2C, S11, and S16). The corresponding proline-containing cofactor **22b** exhibited only a marginal increase in half-life compared to its homoalanine counterpart cofactor **37b**, consistent with their similar depurination rate constants of the order of 10^–5^ s^–1^ (Figures S11 and S17, Table 1). Incorporation of a 7-deazapurine (cofactor **26b**) did not improve stability, likely due to increased lability of the hex-2-ynyl group, resulting in its intramolecular or intermolecular transfer onto proline nitrogen (Table S1, Figures S17, and S18). However, the selenonium-proline cofactor **27b** displayed significantly improved stability of ∼14 h, with degradation predominantly occurring through hydrolysis of the transferable group (Figures S17 and S18). This represents a 12-fold increase in half-life compared to the conventional homoalanine-based analogue **37b** (72 min).

Both the sulfonium proline cofactor **32b** and AdoYn^3,23^ were highly unstable and rapidly degraded via hydration of the terminal alkyne, with complete decomposition occurring in under 1 min (Table S1). The cofactor **40b** (SeAdoYn) is widely used for tagging proteins, DNA, or RNA with a reactive propargyl group for downstream labeling by click chemistry.^13,23,24^ We determined the half-life of this cofactor to be approximately 15 min due to very fast intramolecular cyclization (Figures S16 and S18). In comparison, the selenonium-proline cofactor **33b** exhibited improved stability, with a half-life of ∼45 min (Figures S11 and S17 and Table 1) and degraded via water addition to the triple bond, thus significantly slower compared to AdoYn or **32b**. Introduction of proline eliminated the intramolecular cyclization pathway but did not change susceptibility to water addition at the triple bond. We propose that selenonium salts possess a substantially higher activation barrier to ylide formation at the propargyl substituent, a process that in sulfonium analogues rapidly triggers rearrangement to a highly reactive allene intermediate that is readily attacked by water.

An alternative strategy to overcome propargyl cofactor instability is to position the terminal alkyne distal to the chalcogenonium center.^5^ Thus, we designed cofactor **35b**, featuring a transferable n-octadiynyl group—an eight-carbon linker terminating in a terminal alkyne, which can be used via click chemistry for 2-step labeling or pulldown applications. This design retained a half-life of ∼15 h and degradation kinetics similar to those of cofactor **27b**, which contains a shorter hex-2-ynyl side chain, and showed no degradation via water addition to the triple bond (Figure S17).

### Enzymatic Activity with Methyltransferases TaqI and HhaI Q82A/Y254S/N304A

The efficiency of enzymatic transfer of methyl or triple-bond-activated substituents (R groups) was estimated using a DNA protection assay by varying the molar ratio of DNA methyltransferase to its target sites. Reactions contained a constant amount of λ DNA and 2-fold serial dilutions of M.TaqI, and apparent turnovers were determined based on digestion with R.TaqI, followed by agarose gel electrophoresis (Figures 2D, S6, and S19). Consistent with previous studies, AdoMet showed 512 h^–1^ turnovers and served as a benchmark.^3^ The analogue cofactor **28ab**, bearing a proline side chain, exhibited only 8 h^–1^. This might be caused by multiple reasons, including decreased affinity of the cofactor due to the loss of contacts with the protein, steric hindrance, and distorted geometry of the transition state due to conformational rigidity of the proline side chain. Remarkably, selenonium analogue **30ab** with the same proline side chain reached ∼512 h^–1^, suggesting that the enhanced electrophilicity of selenium and markedly increased cofactor stability can effectively counteract all mentioned negative effects. The same trend was observed for triple-bond-activated cofactor series: the benchmark homoalanine cofactor (**37b**) with the hex-2-ynyl transferable group attained ∼10 h^–1^, while the *cis*-l/d-proline analogues **22b**/**24b** showed reduced activity (∼4 h^–1^). The selenonium-proline cofactor **27b** exhibited 32 h^–1^ turnovers, representing a significant improvement over homoalanine cofactor **37b**. Importantly, this effect was not limited to short transferable chains but was retained in fluorescent cofactors, where the fluorescent dye is linked via a long hydrophilic PEG4 linker. Here again, selenonium-proline cofactor **42** significantly outperformed sulfonium-proline cofactor **41** and homoalanine cofactor **44**. In fact, M.TaqI activity with cofactor **42** approached its activity with AdoMet (Figure 2D). Longer alkyl carbon chain-substituted cofactors, such as -propyl, without activating π-bonds generally result in minimal (<1 h^–1^) transfer efficiencies.^3,25^ Nevertheless, the most stable analogue **31b** (half-life ∼ 50 days), bearing a hexyl transferable group, achieved ∼8 h^–1^ turnovers with M.TaqI, underscoring the increased reactivity of selenonium centers even without sp or sp^2^ hybridization at the β-position^20^ (Figures 2D and S19).

A similar trend was observed with M.HhaI Q82A/Y254S/N304A and cofactors with a transferable methyl group. The activity of proline cofactor **28ab** decreased compared to AdoMet, which was partially rescued using selenonium-proline cofactor **30ab**. However, this effect was not retained with longer transferable chains: while cofactor **22b** showed low activity, cofactor **27b** was completely inactive (Figure S20). This behavior likely results from the stringent geometric requirements for the formation of the intermediate covalent complex in C5-MTases, which cannot simultaneously accommodate the constrained proline side chain and the larger selenium atom.

Next, we confirmed the nature of enzymatic reaction products in the MTase reaction under single-turnover conditions using duplex DNA oligonucleotide bearing a single MTase site and an excess of enzyme and cofactor. The DNA was then hydrolyzed to nucleosides, and LC/MS analysis was used to identify the resulting modifications. Expected identities of modification products by M.TaqI (Figures S21 and S22) and M.HhaI Q82A/Y254S/N304A (Figure S23) were confirmed by mass spectrometry in all the cases. The results corroborate the conclusion that both DNA MTases can use stabilized *cis*-l-proline sulfonium and selenonium cofactors to modify DNA.

Under single-turnover conditions, M.TaqI reaction efficiency is unlikely to be limited by affinity toward the cofactor. Nonetheless, sulfonium-proline cofactors **22b**, **24b**, and **41** and selenonium-proline cofactors **31b** and **43**, which lack activating π bonds, fail to modify both DNA strands. This suggests a challenge in accommodating the conformationally rigid cofactor and the bulky modification of the complementary strand of the DNA target site in a productive orientation within the enzyme active site.

Examination of binary M.TaqI–AdoMet (PDB ID 2ADM) and M.HhaI–AdoMet (PDB ID 2HMY) X-ray structures revealed significant differences in their cofactor binding pockets. The M.TaqI pocket is substantially less sterically constrained than that of M.HhaI, rationalizing its higher tolerance toward analogues (Figure S24 and Videos S1 and S2). Introduction of the Q82A/Y254S/N304A mutations into the M.HhaI active site expands the transferable group cavity while largely preserving interactions with the homoalanine moiety, further supporting the idea that pocket sterics govern cofactor promiscuity (Figure S24).

### Two-Step and Direct Labeling of DNA with Fluorescent Dyes

To demonstrate the applicability of proline-based cofactors for DNA labeling, we carried out two- and single-step sequence-specific labeling of pUC19 DNA (Figure 3). In the two-step approach, proline cofactor **35b**, bearing an n-octadiynyl transferable group, was used. In the first step, the terminal alkyne-containing group was enzymatically transferred to pUC19 DNA by M.TaqI, and complete modification of TCGA sites was confirmed by protection from R.TaqI cleavage (Figure S25). In the second step, the resulting alkyne-tagged DNA was conjugated with Calfluor647-azide^26^ under CuAAC conditions. To assess labeling specificity, the modified DNA was digested with R.MbiI and analyzed by agarose gel electrophoresis. Fluorescence imaging in the Cy5 channel revealed selective labeling of the 1801 and 644 bp fragments—each containing two M.TaqI sites and no labeling on the 240 bp fragment without M.TaqI sites (Figure 3A).

**Figure 3:**
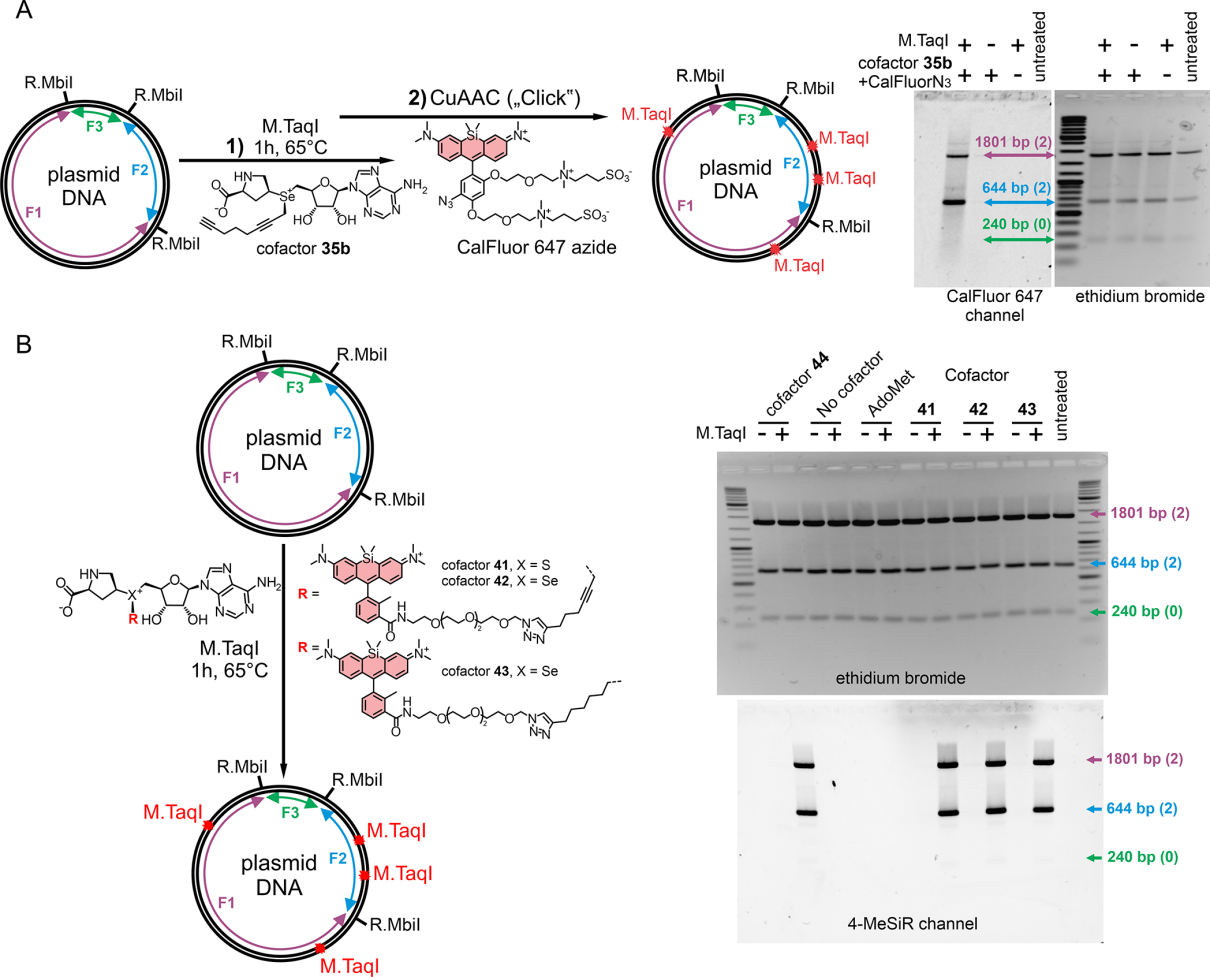
DNA labeling with proline-based AdoMet analogues. Principal scheme of two-step (A) and single-step (B) labeling of pUC19 plasmid (left) and agarose gel electrophoresis demonstrating site-specific DNA labeling after digestion with R.MbiI.

Upon labeling pUC19 DNA with M*.*TaqI and fluorescent proline cofactors, **41**, **42**, and **44** afforded complete protection from R.TaqI digestion, confirming successful fluorophore transfer (Figure S26). The specificity of labeling was validated by digestion with R.MbiI and analyzing fluorescent DNA fragments on agarose gel (Figure 3B). The identity of adenine modifications was confirmed by LC-MS analysis using a duplex oligonucleotide under single-turnover reaction conditions (Figure S27).

## Conclusions

We systematically evaluated all stereoisomers of proline-based AdoMet analogues to define the structural determinants of the cofactor activity. Only the *cis*-l and *cis*-d configurations were catalytically competent, with *cis*-l displaying the highest activity, whereas both trans isomers were inactive. Proline substitution suppressed intramolecular cyclization but had a minimal impact on depurination kinetics. Depurination was markedly reduced upon replacement of sulfonium with selenonium, extending cofactor half-lives by up to 90-fold without compromising transfer rates.

The improved cofactor stability significantly enhances the practical utility of methyltransferase-mediated tagging. In particular, efficient labeling is possible under the elevated temperatures required for thermophilic methyltransferases. This design strategy paves the way for more robust biomolecular labeling tools, and we believe it may be extended to other types of AdoMet-dependent enzymatic systems. The modularity of the approach also supports future development of stable and specifically tailored AdoMet analogues for tagging, epigenetic profiling, or other applications in diagnostics.

## Supplementary Material







## Notes

Figures, tables, experimental procedures, NMR spectra, and HPLC chromatograms (PDF)
Rotating view of the binary M.TaqI–AdoMet complex (PDB ID 2ADM) highlighting cofactor positioning and binding pocket (MP4)
Rotating view of the binary M.HhaI–AdoMet complex (PDB ID 2HMY) illustrating cofactor positioning within the binding pocket (MP4)
